# *Plasmodium falciparum* UvrD activities are downregulated by DNA-interacting compounds and its dsRNA inhibits malaria parasite growth

**DOI:** 10.1186/1471-2091-15-9

**Published:** 2014-04-03

**Authors:** Mohammed Tarique, Farha Tabassum, Moaz Ahmad, Renu Tuteja

**Affiliations:** 1 International Centre for Genetic Engineering and Biotechnology, P. O. Box 10504, Aruna Asaf Ali Marg, New Delhi 110067, India

**Keywords:** ATPase, Helicase, Malaria parasite, UvrD, DNA unwinding, DNA interacting agents

## Abstract

**Background:**

Human malaria parasite infection and its control is a global challenge which is responsible for ~0.65 million deaths every year globally. The emergence of drug resistant malaria parasite is another challenge to fight with malaria. Enormous efforts are being made to identify suitable drug targets in order to develop newer classes of drug. Helicases play crucial roles in DNA metabolism and have been proposed as therapeutic targets for cancer therapy as well as viral and parasitic infections. Genome wide analysis revealed that *Plasmodium falciparum* possesses UvrD helicase, which is absent in the human host.

**Results:**

Recently the biochemical characterization of *P.* f*alciparum* UvrD helicase revealed that N-terminal UvrD (PfUDN) hydrolyses ATP, translocates in 3’ to 5’ direction and interacts with MLH to modulate each other’s activity. In this follow up study, further characterization of *P. falciparum* UvrD helicase is presented. Here, we screened the effect of various DNA interacting compounds on the ATPase and helicase activity of PfUDN. This study resulted into the identification of daunorubicin (daunomycin), netropsin, nogalamycin, and ethidium bromide as the potential inhibitor molecules for the biochemical activities of PfUDN with IC_50_ values ranging from ~3.0 to ~5.0 μM. Interestingly etoposide did not inhibit the ATPase activity but considerable inhibition of unwinding activity was observed at 20 μM. Further study for analyzing the importance of PfUvrD enzyme in parasite growth revealed that PfUvrD is crucial/important for its growth ex-vivo.

**Conclusions:**

As PfUvrD is absent in human hence on the basis of this study we propose PfUvrD as suitable drug target to control malaria. Some of the PfUvrD inhibitors identified in the present study can be utilized to further design novel and specific inhibitor molecules.

## Background

Human malaria is the parasitic disease caused by five different species of Plasmodium *(Plasmodium falciparum, P. vivax, P. ovale, P. malarie and P. knowlesi),* among them *P. falciparum* is responsible for the most severe and lethal infection (reviewed by [1]). There is controversy over the number of human deaths due to malaria but as per WHO report malaria is responsible for ~0.65 million deaths every year [2,3]. The efforts to identify suitable drug targets to fight with malaria parasite infections are a global concern [1,4] as many previous attempts have been unsuccessful to develop new class of antimalarial drugs. Similarly the attempts to develop vaccine for malaria are not very promising and a vaccine for malaria is not possible in near future [2,5,6]. There are certainly limited drugs available for treatment of malaria that includes chloroquine, sulphadoxine, pyrimethamine and derivatives. During the last few years the situation has worsened mainly due to the emergence of malaria parasite resistant to several anti-malarial drugs and their spread in other region of the world [7-9]. Thus it is equally important to explore the underlying cause of the emergence of drug resistant parasite and development of novel therapeutics to treat the resistant malaria parasite infections. Although many putative therapeutic targets for malaria have been reported in the recent past [10-19] but further studies are required in order to get the insight for the successful development of suitable drugs. Helicases have also been proposed as suitable drug targets for human cancers as well as many pathogens including viruses and *P. falciparum*[20-33].

Helicases are molecular motor proteins that play important roles in the metabolism of nucleic acids. It is well established that in-vivo each helicase is specialized for its own helicase activity led biochemical role. The genome wide analysis of *P. falciparum* helicases revealed that the parasite contains a UvrD helicase while this enzyme is absent in human host [34,35]. Later, the biochemical characterization of this *P. falciparum* specific UvrD helicase revealed its characteristic biochemical activities [32].

UvrD helicase has been characterized from *Escherichia coli* and *Mycobacterium tuberculosis*[36-38] and it is a critical component of the DNA repair process of prokaryotes. In particular, UvrD is required for the mismatch repair (MMR) and nucleotide excision repair (NER) to repair mismatches occurring during replication or DNA lesions such as UV-induced pyrimidine dimers or more bulky adducts [35,39,40]. In a recent study we have reported the detailed biochemical characterization of the UvrD helicase from *P. falciparum* and have shown that it exhibits the ssDNA-dependent ATPase activity and the DNA helicase activity in 3’ to 5’ direction [32,33,35]. Recent study on UvrD in different Plasmodium species revealed that the other species of Plasmodium also contain putative UvrD helicase [33].

Here, in the follow up study of our previous work [32], we report the effect of different DNA interacting compounds (actinomycin, camptothecin, ciprofloxacin, cisplatin, cyclophosphamide, DAPI, daunorubicin, etoposide, ethidium bromide, genistein, morin, netropsin, nogalamycin and novobiocin) on the helicase and ATPase activities of PfUvrD helicase. Out of all these molecules tested, only daunorubicin, ethidium bromide, netropsin, and nogalamycin were found to be potent inhibitors for the PfUDN enzymatic activities. The inhibition of PfUvrD by dsRNA showed that it is required for the ex-vivo intraerythrocytic development of the parasite *P. falciparum* 3D7. This study will set the stage for designing the specific inhibitors for inhibiting the enzymatic activity of PfUDN which in turn will block the parasite growth as well.

## Methods

### Materials

Nucleoside triphosphates and deoxynucleoside triphosphates were obtained from Pharmacia (Uppsala, Sweden) and [γ-^32^P] ATP was purchased from Perkin Elmer (Boston, MA, USA). M13mp19 ssDNA was purchased from Invitrogen (Carlsbad, CA, USA). Synthetic DNA oligonucleotides were synthesized chemically. The DNA-interacting compounds, camptothecin, ciprofloxacin, cisplatin, DAPI, daunorubicin, etoposide and morin were purchased from Topogene Inc. (Columbus, OH, USA). Cyclophosphamide, genistein, netropsin, nogalamycin and novobiocin were obtained from Sigma Chemical Co. (St Louis, MO, USA), ethidium bromide and actinomycin was obtained from BDH (E. Merck, Mumbai, India) and Boehringer Mannheim (Indianapolis, IN, USA), respectively. All of these compounds were dissolved in dimethylsulfoxide (DMSO) and stored at 4°C in the dark.

### DNA dependent ATPase assay

Standard protocol of ATPase assay was followed as described in the previous report [32,41]. The hydrolysis of ATP catalyzed by PfUDN was assayed by measuring the formation of Pi from [γ-^32^P] ATP. The reaction mixture of 10 μl contained [γ-^32^P] ATP (specific activity 222 TBq.mmol^-1^) and cold ATP (1 mM), ATPase buffer (20 mM Tris-HCl, pH 8.0, 8 mM DTT, 1.0 mM MgCl_2_, 20 mM KCl and 16 μg/ml BSA), purified PfUDN and 50 ng or 100 ng of M13 mp19 ssDNA. The reaction mixtures were incubated at 37°C for 60 min. This was followed by thin layer chromatography (TLC), and the quantitation was done using Alpha Imager-EP/Image-J software ( http://rsbweb.nih.gov/ij/). In order to study the effect of various compounds on ssDNA-dependent ATPase activity, different compounds were added into the reaction mixture prior to the addition of the PfUDN. In another set of experiment, PfUDN was pre-incubated (15 min) with these compounds in order to obtain the insight of inhibitory mechanism. All the experiments were performed at least two times and quantification data was used to calculate the standard deviation using Microsoft Excel 2010 and mean value was used to prepare the graph.

### Preparation of substrate and DNA helicase assay

Helicase assay was demonstrated using the purified fraction of PfUDN. The specially designed partial duplex substrate consisted of a ^32^P-labelled 47-mer DNA oligodeoxynucleotide annealed to M13mp19 phage ssDNA. This oligodeoxynucleotide of the nucleotide sequence 5'- (T)_15_GTTTTCCCAGTCACGAC(T)_15_-3' contains 15 base-pairs of non-complementary region (T)_15_ at both the 5’ and 3’ ends. Oligodeoxynucleotide was labeled at 5′-end with T4 polynucleotide kinase (PNK) (5U) (New England Biolabs) and 1.85 MBq of [γ-^32^P] ATP (specific activity 222 TBq/mmol) at 37°C for one hour and then annealed using standard annealing buffer (20 mM Tris-HCl, pH 7.5, 10 mM MgCl_2_, 100 mM NaCl, 1 mM DTT) with 0.5 μg of single-stranded circular M13mp19 (+) phage DNA by heating at 95°C for 1 min and then transferring immediately to 65°C for 2 min and then slow cooling to room temperature. Using gel filtration through a Sepharose 4B column (Pharmacia, Sweden) the non-hybridized oligodeoxynucleotide was removed [32]. The reaction volume of 10 μl containing the ^32^P-labeled helicase substrate (1000 cpm/10 μl) in appropriate buffer (20 mMTris-HCl, pH 8.0, 8 mM DTT, 1.0 mM MgCl_2_, 20 mMKCl and 16 μg/ml BSA) and PfUDN was incubated at 37°C for 60 min. The substrate and products were separated by electrophoresis on a nondenaturing 12% PAGE and the gel was exposed to hyper film for autoradiography or scanned on phosphoimager. In order to study the effect of DNA-interacting compounds on helicase activity, different compounds were added to the helicase reaction mixture prior to the addition of the PfUDN helicase. All the experiments were performed in duplicate and quantitation of both the substrate and unwound DNA bands was done using AlphaImager-EP/Image-J software ( http://rsbweb.nih.gov/ij/). Quantification data was used to calculate the standard deviation using Microsoft Excel 2010 and mean value was used to prepare the graph.

### Double stranded (ds) RNA preparation

The clone of PfUDN in the pGEMT easy vector of Promega (Madison, WI, USA) was used as a template to amplify the gene, using T7 and SP6 primers [42]. Similarly the clone of GFP into pGEMT easy vector was used as control. The PCR products using these two primers were purified using the Wizard DNA clean-up system from Promega. These purified templates were used for in vitro transcription to generate sense RNA (sRNA) and anti-sense RNA (asRNA) using T7 and the SP6 RiboMAX Express large-scale RNA production system from Promega. For the production of dsRNA, equal amounts of sRNA and asRNA were mixed and incubated at 65°C for 30 min, and then the incubation was continued at room temperature overnight. The mixture was treated with DNase and precipitated after phenol/chloroform extraction. The pellet of dsRNA was dissolved in diethyl pyrocarbonate water and treated with RNase T1. These samples were checked on 1% (w⁄v) native agarose gel. This dsRNA was quantitated and used for the following experiments.

### Parasite proliferation assay in presence of dsRNA targeted to UvrD helicase

For analyzing the effect of dsRNA, the cultures were adjusted to 4% hematocrit with 1% infected red blood cells (RBCs). 200 μl of this mixture was centrifuged at 1500xg in a Sorvall RT7 centrifuge (Du Pont, Newtown, CT, USA), and the pellet was resuspended in 50 μl of incomplete medium and 20 μg dsRNA per ml was added to this mixture. This mixture was incubated at 37°C with intermittent mixing to avoid settling of RBCs. After this incubation, serum was added to a final concentration of 20% and the mixture was dispensed in 96-well plates and incubated at 37°C for specific times. The smears were made at different intraerythrocytic developmental stage of the parasite and the effect was determined by microscopic examination as well as cyber green based assay [43]. The parasite proliferation assay was performed in duplicate and quantification data (using ELISA reader) was used to calculate the standard deviation using Microsoft Excel 2010 and mean value was used to prepare the bar diagram.

## Results

### Effect of various compounds on ssDNA-dependent ATPase activity

**Figure 1:**
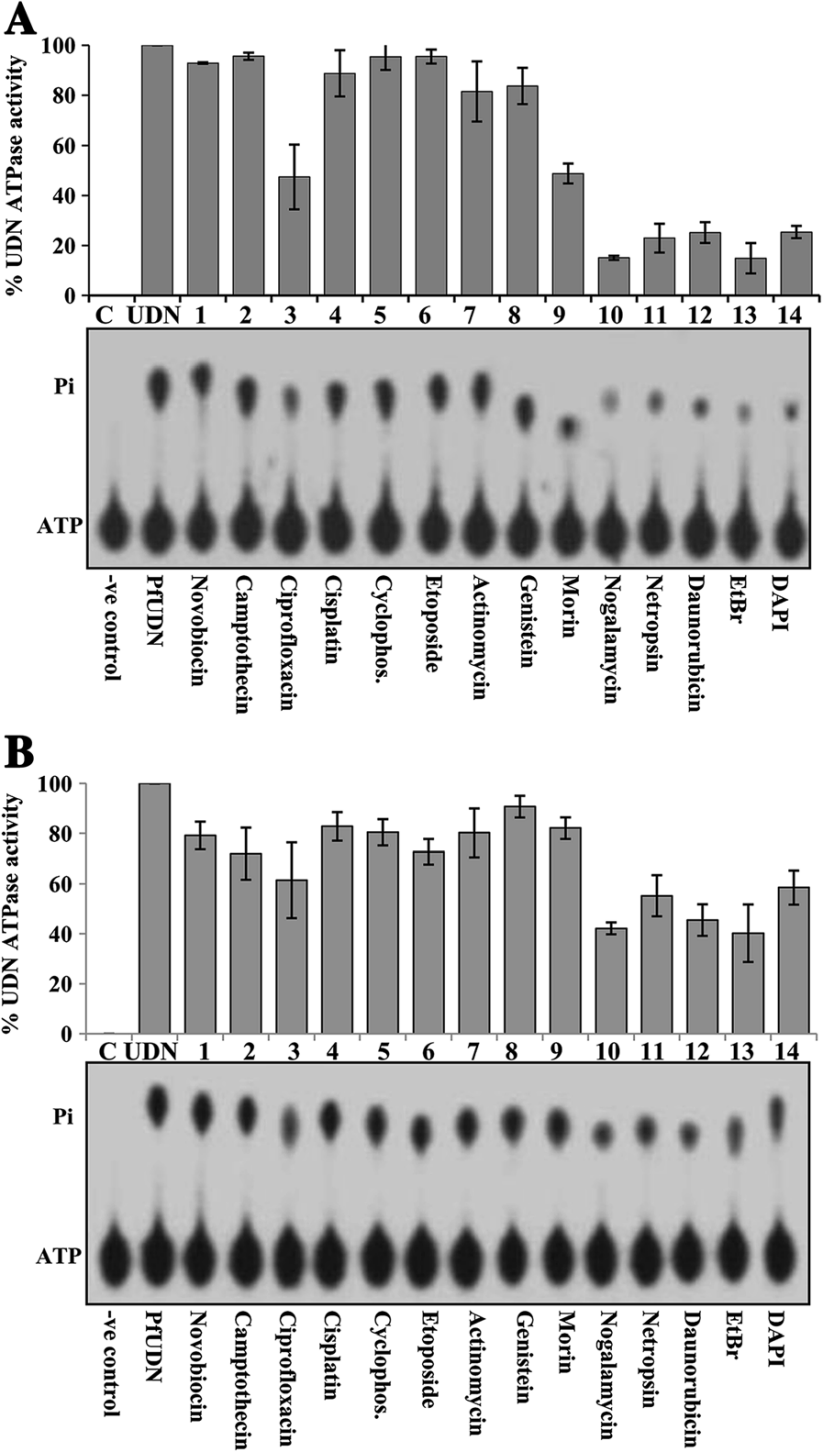
**Effect of various compounds (20 μM) on the ATPase activity. A**. ATPase assay using DNA preincubated with the compounds. **B**. ATPase assay using protein preincubated with the compounds. Percent ATPase activity of PfUDN in the presence of various compounds is presented in the bar diagram corresponding to the autoradiogram. Lane C is no enzyme control, lane UDN is control reaction of PfUDN without the addition of compound. Lanes 1-14 are the ATPase reactions with enzyme in the presence of different compounds labeled below the autoradiogram. The position of ATP and released Pi is shown on the left side of the autoradiogram.

**Figure 2:**
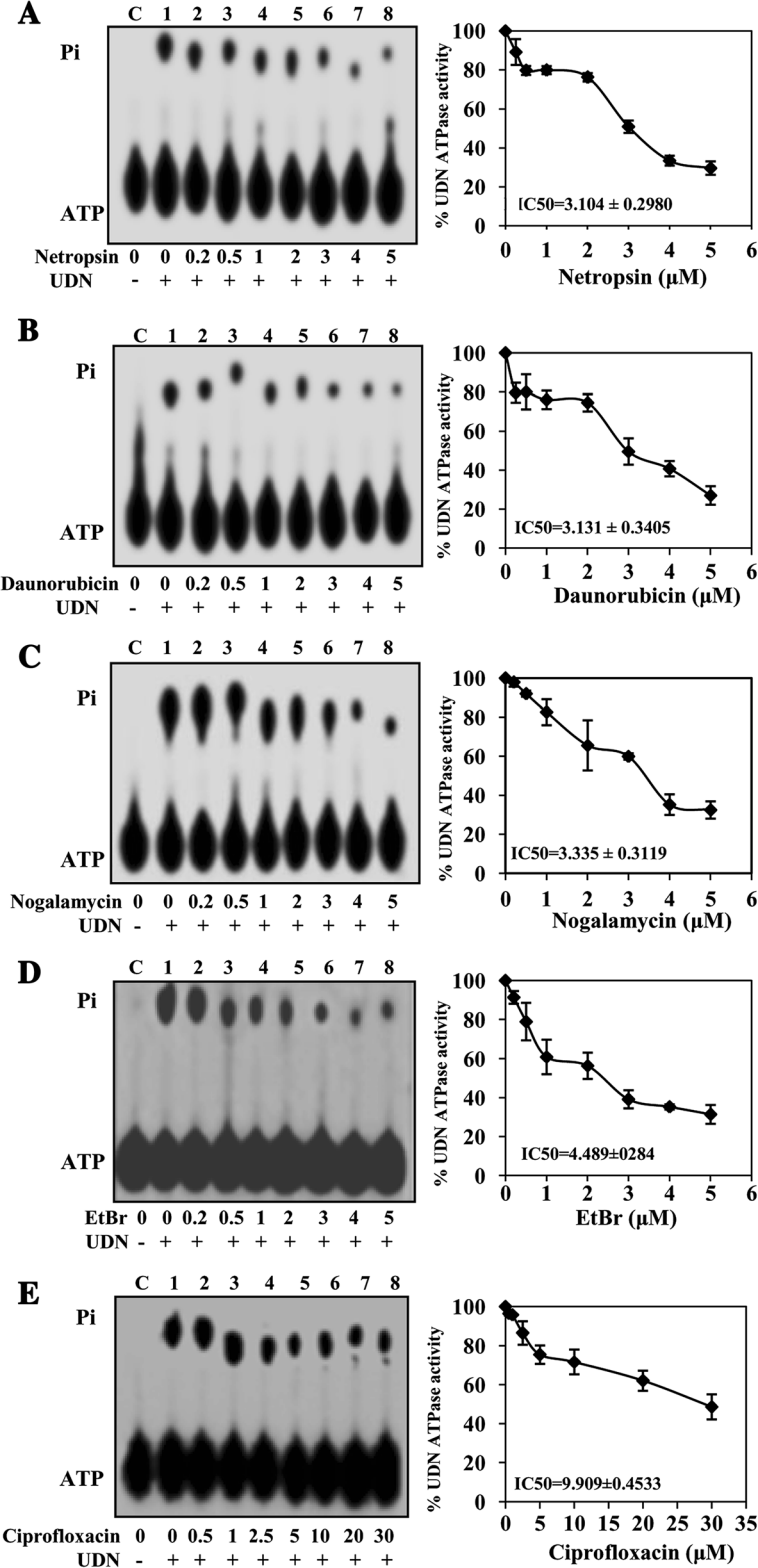
**Analysis of ATPase inhibition kinetics.** Concentration curves of compounds **A**. Netropsin, **B**. Daunorubicin, **C**. Nogalamycin, **D**. ethidium bromide and **E**. Ciprofloxacin. In each panel, the autoradiogram of the assay is shown on the left and the quantitative data on the right side. Lane C in all the panels is control without enzyme and lane 1 in all the panels is reaction with enzyme without the addition of any compound. Lanes 2–8 are reactions with enzyme in the presence of 0.2, 0.5, 1, 2, 3, 4 and 5 μM compounds labeled below the autoradiogram. Inhibition kinetics of ciprofloxacin was performed using 0.5-30 μM concentration. The position of ATP and released Pi is shown on the left side of the autoradiogram.

The effect of different compounds (20 μM) on the ssDNA dependent ATPase activity of PfUDN (100 nM) was studied using the protocol described in the material and methods. In the first set of experiment (Figure 1A), reaction mixture was preincubated (15 min) with the compounds (20 μM) and then PfUDN was added to the mixture. The results clearly show that ciprofloxacin, morin, nogalamycin, netropsin, daunobrubicin, ethidium bromide and DAPI, (Figure 1A, lanes 3, 9-14 respectively) inhibit the ssDNA dependent ATPase activity of PfUDN under these conditions. Among these compounds, which inhibited the ATPase activity, nogalamycin, daunorubicin, netropsin, and ethidium bromide are the top four inhibitors of ATPase activity at 20 μM concentration. Ciprofloxacin and morin weakly inhibit the ATPase activity of PfUDN (Figure 1A, lanes 3 and 9, respectively). The other compounds like novobiocin, camptothecin, cisplatin, cyclophosphamide, etoposide, actinomycin, and genistein were not able to considerably inhibit the ssDNA dependent ATPase activity of PfUDN at 20 μM concentration (Figure 1A, lanes 1, 2 and 4-8, respectively). In the second set of experiment, PfUDN was preincubated with the compound (15 min) then the reaction mixture was added. The results show that the intensity of inhibition was considerably decreased (Figure 1B, lanes 3, 9-14 respectively). Thus from these observations it seems that these compounds (daunorubicin, netropsin, nogalamycin, ethidium bromide, morin and ciprofloxacin) potentially inhibited the ATPase activity possibly by interfering with the DNA binding of PfUDN protein. To get further insight another assay was performed with fixed concentration of inhibitor molecule but in the presence of 100 ng of ssDNA in the reaction and the results show that inhibition was slightly less as compared to the reactions containing 50 ng ssDNA in the reaction (Additional file 1: Figure S1). These compounds (daunorubicin, netropsin, nogalamycin, ethidium bromide and ciprofloxacin) were further used for the study of kinetics of inhibition of PfUDN ATPase activity. Increasing concentration of inhibitors (0.2 to 5.0 μM) was used in the ATPase reaction (Figure 2A- 2D, lanes 1-8 in each panel) and the results show that the effective inhibitors are netropsin, daunorubicin, nogalamycin and ethidium bromide with IC_50_ value ranging from ~3.0 to ~4.5 μM, respectively (Figure 2A- 2D) (Table 1). It is interesting to note that ciprofloxacin was effective only at higher concentration and the IC_50_ value for ciprofloxacin is ~10.0 μM (Figure 2E, lanes 2-8) (Table 1).

**Table 1:** Comparison of inhibitory potential for ATPase activity

**Compounds**	**PfUDN**	**PfH45**	**PfD66/PfDDX19**	**PfDH60**
Ciprofloxacin	9.9	ND	ND	ND
Ethidium bromide	3.1	1.5	1.5	2.5
Daunorubicin	4.4	5.0	ND	3.0
Netropsin	3.1	1.5	1.0	3.0
Nogalamycin	3.3	0.8	3.2	0.5

ND- Not determined.

### Effect of various compounds on DNA helicase activity of PfUDN

**Figure 3:**
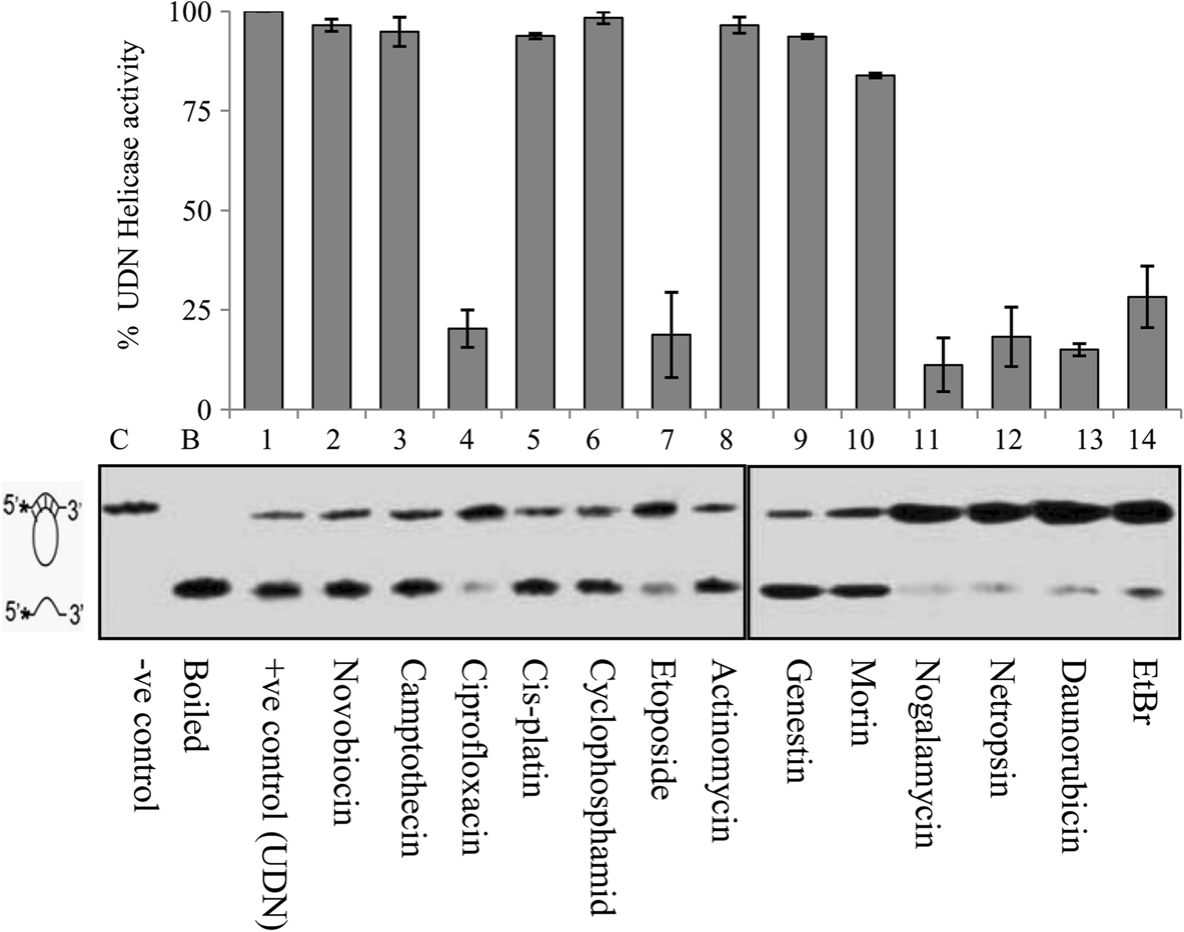
**The effect of different compounds on the DNA unwinding activity of PfUDN.** The structure of the DNA helicase substrate is shown on the left side of the autoradiogram of the gel. Lane C is the control without enzyme and lane B is heated substrate. Lane 1 is the control reaction with enzyme without any added compound. Lanes 2–14 are the reactions with enzyme in the presence of different compounds (20 μM) labeled below the autoradiogram. Percent helicase activity of PfUDN in presence of various compounds are presented in the bar diagram corresponding to the autoradiogram.

The DNA helicase or unwinding activity of PfUDN in the presence of 20 μM of different compounds was tested separately by using the partial duplex substrate. This duplex substrate used in the helicase reaction, has 15 mer overhangs (i.e. non complementary region) on both the 5’ and 3’ends and 17 nucleotide region annealed with the circular DNA. The results obtained clearly show that all the compounds which inhibited the ATPase activity i.e. ciprofloxacin, nogalamycin, netropsin, daunorubicin and ethidium bromide also inhibit the DNA unwinding activity of PfUDN effectively with different strength (Figure 3, lanes 4, and 11-14 respectively). Interestingly etoposide, which does not inhibit the ATPase activity, blocks the helicase activity appreciably at 20 μM under in vitro conditions (Figure 3, lane 7). Whereas the other nucleic acid binding agents like novobiocin, camptothecin, cisplatin, cyclophosphamide, actinomycin, genistein and morin were not able to considerably inhibit the DNA unwinding activity of PfUDN in in vitro conditions (Figure 3, lanes 2, 3, 5, 6, 8-10, respectively). These inhibitor molecules (daunorubicin, netropsin, nogalamycin and ethidium bromide) were further used for the kinetic study of helicase activity of PfUDN.

**Figure 4:**
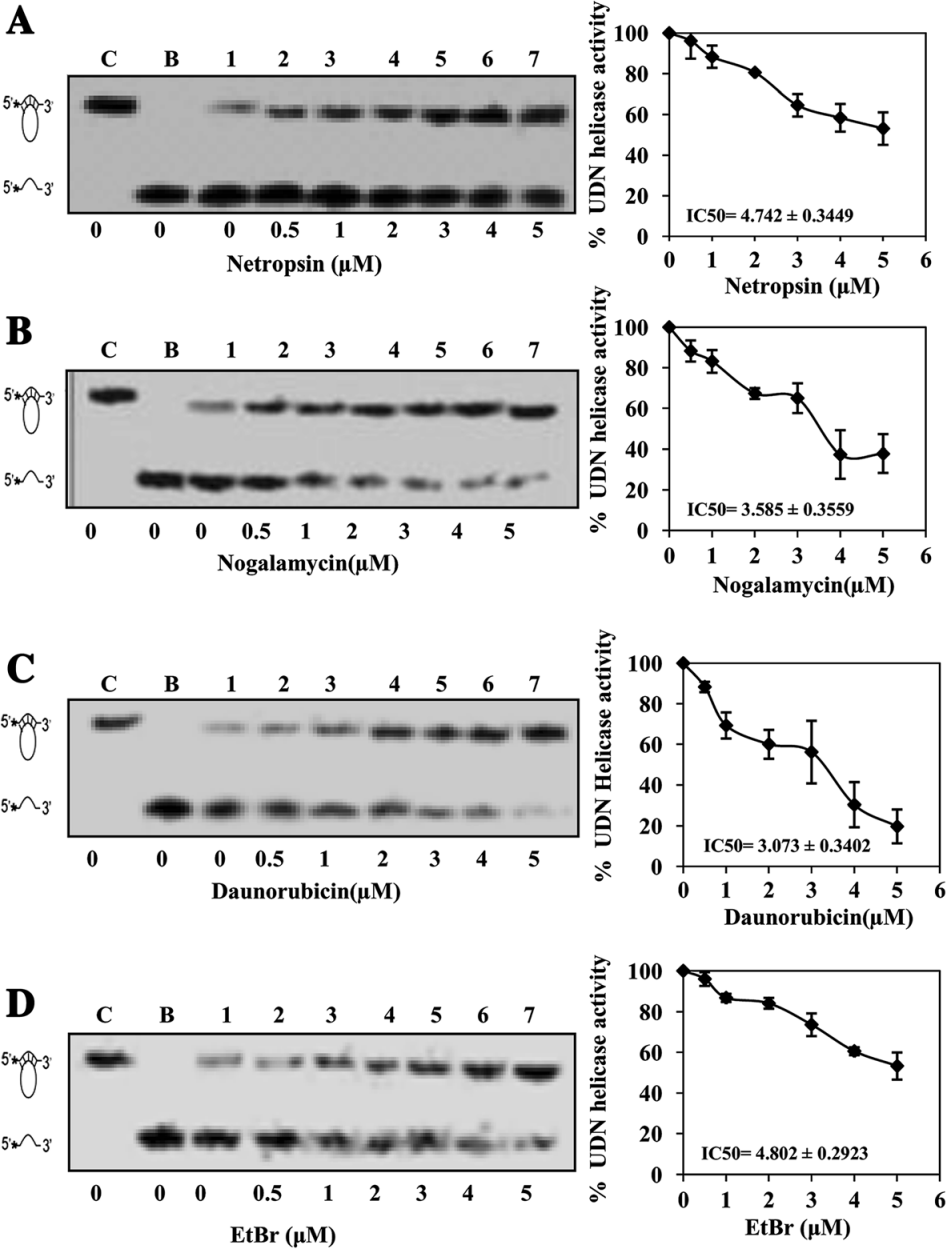
**Analysis of helicase inhibition kinetics.** Concentration curves of compounds **A**. netropsin, **B**. nogalamycin, **C**. daunorubicin, and **D**. ethidium bromide. In each panel, the autoradiogram of the assay is shown on the left and the quantitative data on the right side. Lane C in all the panels is control without enzyme and lane B in all the panels is heated substrate. Lanes 1-7 are reactions with enzyme in the presence of 0, 0.5, 1, 2, 3, 4 and 5 μM of compounds labeled below the autoradiogram.

The concentration of inhibitors used in unwinding reaction of PfUDN ranged from 0.5-5.0 μM and the results show clearly that the effective inhibitor is daunorubicin with IC_50_ value of ~3.0 μM (Figure 4C, lanes 2-7) (Table 2), followed by other inhibitors such as nogalamycin (Figure 4B, lanes 2-7), netropsin (Figure 4A, lanes 2-7) and ethidium bromide (Figure 4D, lanes 2-7) with IC_50_ values ranging from ~3-5 μM, respectively (Table 2). Thus these results clearly show that these four compounds have potential to inhibit the helicase activity of PfUDN. Although etoposide and ciprofloxacin inhibited the helicase activity at higher (20 μM) concentration but these compounds failed to show appreciable inhibition upto 5 μM concentration tested (data not shown).

**Table 2:** Comparison of inhibitory potential for helicase activity

**Compounds**	**PfUDN**	**PfH45**	**PfD66/PfDDX19**	**PfDH60**
Ethidium bromide	4.8	1.0	1.0	1.5
Daunorubicin	3.0	1.5	ND	0.3
Netropsin	4.7	0.5	0.5	1.2
Nogalamycin	3.5	0.5	5.0	2.0

ND- Not determined.

### Inhibition study of parasite growth by PfUDN ds-RNA

**Figure 5:**
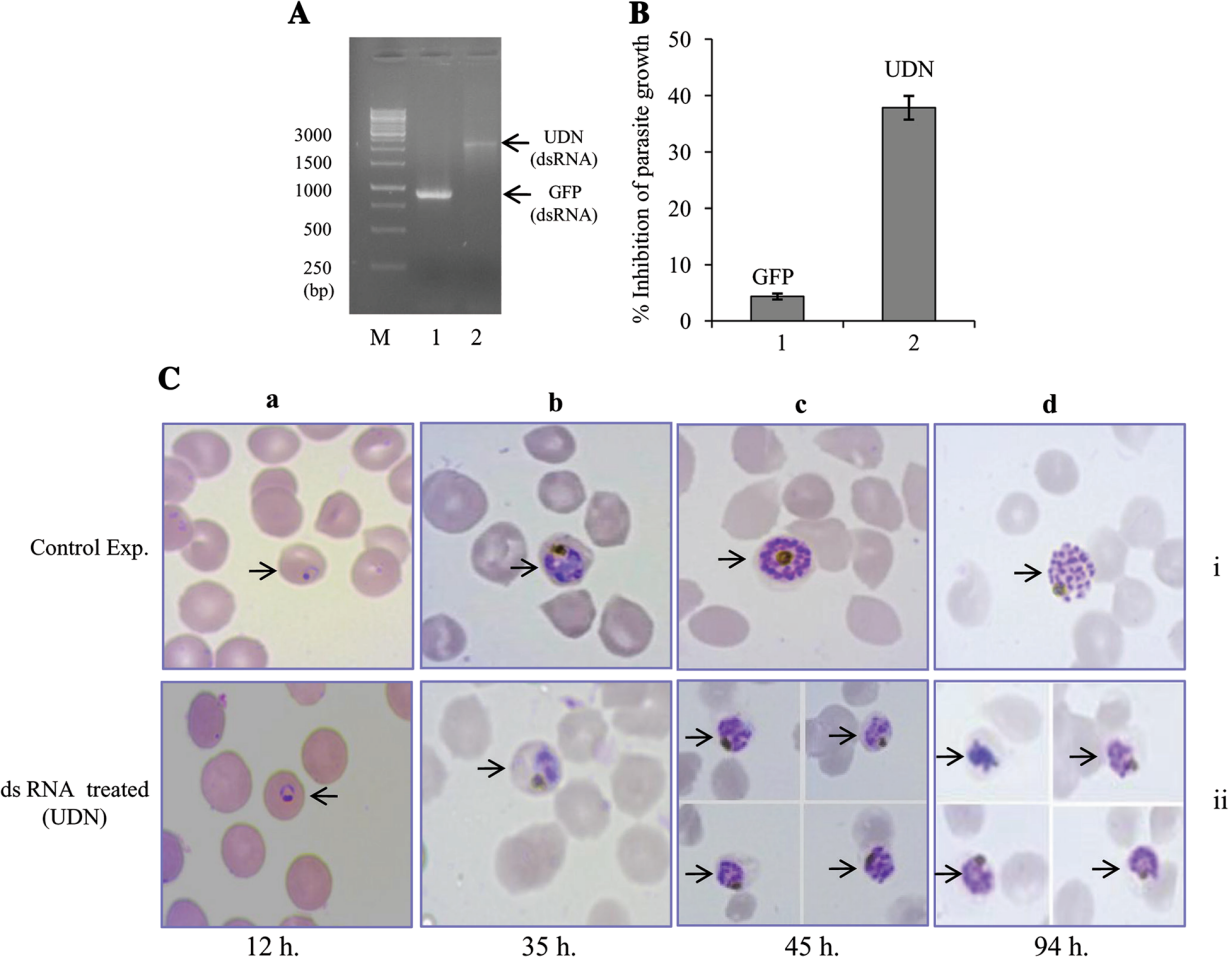
**Effect of dsRNA on the *ex-vivo *growth of the *Plasmodium falciparum *within red blood cells.** Parasite growth evaluated by the SYBER green assay using synchronized cultures. Figure **A**. shows ds RNA preparation of green fluorescent protein (GFP) (lane 1) and PfUDN (lane 2) lane M is the marker. **B**. shows the growth of culture 50 hour post dsRNA treatment (i.e. at 56 hour stage of parasite growth) with the dsRNA of GFP (lane 1 in A), and PfUDN (lane 2 in A). **C**. Giemsa-stained parasite infected RBC after dsRNA treatment. Control panel in (i) shows the culture after treatment with GFP-dsRNA while panel (ii) shows the culture after treatment with dsRNA of PfUDN at different time interval as labeled. The arrows in each panel show *P. falciparum* infected RBC at different developmental stages.

Previously it has been shown that dsRNA based knockdown of gene(s) worked effectively at least for the DNA helicases of *P. falciparum*[42]. The P*. falciparum* 3D7 culture was treated with PfUDN dsRNA (Figure 5A) (20 μg/ml) in synchronized culture and the dsRNA of green fluorescent protein (GFP) gene was used as control in this study. It is interesting to note that the parasite growth was inhibited ~40% (at 56 hour stage i.e. ring stage) by the addition of PfUDN dsRNA in culture (Figure 5B lane 2). The treated as well as control culture was monitored at different time interval at all the developmental stages using Giemsa staining. The result of control experiments (Figure 5C (i) in a-d), revealed that the morphology of the parasite developmental stage is almost normal. The morphology of the parasite is less distorted after the treatment with PfUDN dsRNA alone during ring and trophozoite stage (Figure 5C (ii) in a and b), while during the late trophozoite to schizont stages considerable inhibition was noticed at both first and second cycle of asexual development (Figure 5C (ii) in c and d). The results clearly show that PfUDN dsRNA inhibits the parasite growth especially during early schizont stage, which is evidenced from morphological changes during *ex-vivo* growth of the parasite. It seems that proper nuclear division during schizont stage is hampered in the PfUDN dsRNA treated cultures and punctate nuclei of schizont stage were rare as compared to the control experiment. Parasitemia at schizont stage of the second cycle was roughly ~8% in control experiment (GFP-dsRNA treated) while parasitemia decreases to 4-5% in PfUDN-dsRNA treated culture. Interestingly, most of the UDN-dsRNA treated parasites were in stress and did not show punctate nuclei at 94 hour stage (schizonts of second cycle). The inhibition of the parasite growth was more prominent at 94-95 hour (second cycle) compared to the 45-46 hour of parasite development (Figure 5C (ii) in d).

## Discussion

The identification of suitable drug target and newer class of antimalarial drugs has been a challenging task for the parasitologist worldwide. In order to consider a parasite protein as a drug target, it should be parasite specific and importantly essential for the parasite growth. In silico data revealed that parasite is unique with respect to the UvrD helicase as its human host lacks this essential enzyme. Furthermore, gene disruption or knockout study is required to firmly establish whether PfUvrD is essential for the malaria parasite growth. Interestingly PfUDN interaction with PfMLH, a crucial MMR protein, and their interplay to regulate each other activity points toward the importance of PfUvrD for the malaria parasite. On the basis of these observations, PfUvrD is expected as crucial helicase for the parasite’s intraerythrocytic developments and was proposed as suitable drug target to combat with dreaded infections of malaria parasite. dsRNA based inhibition study suggests that UvrD is an important enzyme and seems crucial for the successful nuclear division during early schizont stages of *P. falciparum.* Although, mechanism of action of dsRNA is not yet explored and has been controversial in these parasites [44-48].

In order to find the molecules which block the biochemical activities (ATP hydrolyzing and DNA unwinding activities) of PfUvrD, screening with various compounds including DNA interacting compounds was done in this study. The results of this study revealed that daunorubicin inhibited both ATPase and DNA unwinding or helicase activity of PfUDN with IC_50_ value of ~3 μM. Daunorubicin or daunomycin belongs to anthracycline family and is most commonly used as chemotherapeutic for the treatment specifically of acute myeloid leukemia and acute lymphocytic leukemia [49]. Similar to the daunorubicin some other compounds like netropsin, nogalamycin and ethidium bromide also considerably inhibited the DNA dependent ATPase activity of PfUDN at low μM concentration. Previously, the effects of daunorubicin, netropsin and nogalamycin (50 μM) have been tested in the parasite culture (synchronized and unsynchronized) which showed considerable inhibition of parasite growth (~60-70% inhibition at 72 hour) [42]. Similarly, ciprofloxacin has also been found effective for the inhibition of malaria parasite growth [50,51]. Etoposide (VP16) which inhibits DNA unwinding activity of PfUDN, is clinically active against Kaposi’s sarcoma, testicular cancer, acute lymphocytic leukaemia and small-cell lung cancer [52]. It was reviewed that effects of etoposide treatment on *P. falciparum* parasites showed enhanced cleavage of both nuclear and apicoplast and the induction of an apoptosis-like cell death process [52].

The inhibition of the ATPase activity with these compounds was prominent when DNA was preincubated with inhibitor molecule (Figure 1A versus Figure 1B). Thus the results indicate that possibly these compounds interfere with the binding of DNA with PfUDN protein, which ultimately leads to inhibition of DNA dependent ATP hydrolysis. Interestingly all these commercially available molecules, which have been found as inhibitor for the ATPase activity also inhibit the DNA helicase activity of PfUDN helicase. Thus this study further suggests that energy from the ATP hydrolysis is used as driving force for the DNA unwinding activity. Although, ciprofloxacin inhibited the helicase activity during screening at 20 μM concentration but it did not appreciably inhibit the activity at lower concentration (0.5-5 μM). Ciprofloxacin is a fluoroquinolone antibiotic, which moderately inhibited the ATPase as well as helicase activity of PfUDN helicase and it has also been reported to block the helicase activity of Mcm2-7 helicase [53]. Interestingly, in the present study etoposide did not exhibit inhibition on the ATPase activity of PfUDN but significant inhibition of helicase activity was observed at 20 μM concentration. Similar to the ciprofloxacin, etoposide also failed to show appreciable inhibition of the helicase activity of PfUDN at lower concentration (0.5-5 μM).

Ethidium bromide is well known DNA intercalating compound thus it seems that after binding with DNA this compound blocks the interaction of PfUDN with DNA and inhibits the ATPase activity led helicase activity. Although, ethidium bromide cannot be used as drug because of its properties as potential mutagen but other inhibitors reported in the present study can be used as basic molecule to design and synthesize derivatives specific for the PfUvrD helicase. The concentration at which these compounds inhibit the ATPase and helicase activity of PfUDN is certainly not suitable for the therapeutics purpose but this study provides a basic idea to develop novel derivatives in order to achieve significant inhibition of ATPase and helicase activity of PfUvrD at much lower concentration. Thus this study will certainly help pharmacologists to develop derivative of these drugs specific to the PfUvrD which may result into significant inhibition at much lower concentration with the hope to be useful to fight with malaria. Furthermore the knockout out or gene disruption analysis of PfUvrD is required in order to establish the essentiality of UvrD for the parasite, *P. falciparum* but the results of present study with dsRNA targeted to N-terminal of UvrD at least indicates that PfUvrD is crucial for parasite and its growth is inhibited considerably during early schizont stages of asexual life cycle.

## Conclusions

In previous studies we reported the detailed biochemical characterization of malaria parasite *Plasmodium falciparum* specific UvrD helicase [32]. The biochemical studies revealed that PfUvrD exhibits the ATPase as well as DNA helicase activity. It unwinds DNA duplex in 3’-5’ direction and interacts with PfMLH to regulate each other biochemical activities [32]. Recently, we have also reported that the genetically engineered synthetic UvrD helicase (appreciably shorter than native PfUvrD) also showed ATPase and helicase activity [54]. In this study, we have identified daunorubicin, ethidium bromide, netropsin, and nogalamycin as potent inhibitors for the PfUDN enzymatic activities. The results further show that PfUvrD is crucial for the parasite survival as its dsRNA showed inhibition of intraerythrocytic development of the parasite *P. falciparum* 3D7 strain. This study strengthens the speculation that PfUvrD can serve as suitable drug target to control the malaria. Findings of this work can be used for designing the specific inhibitors for PfUvrD, which may result into specific inhibition of UvrD and in turn the parasite growth.

## Abbreviations

ATP: Adenosine triphosphate; dsRNA: Double stranded RNA; MMR: Mismatch repair; NER: Nucleotide excision repair; PfUDN: PfUvrD N terminal.

## Competing interests

The authors declare that they have no competing interests.

## Authors’ contribution

RT wrote the project and received the grant for this study. MT and FT performed the experiments. RT, MA and MT designed the experiments, analyzed the data and wrote the manuscript. All authors read and approved of the final manuscript.

## Supplementary Material

Additional file 1 **Effect of various compounds (20 μM) on the ATPase activity in presence of 100 ng of ssDNA.** ATPase assay using DNA preincubated with the compounds was done. Percent ATPase activity of PfUDN in the presence of various compounds is presented in the bar diagram corresponding to the autoradiogram. C is no enzyme control, lane UDN is control reaction of PfUDN without the addition of compound. Lanes 1-14 are the ATPase reactions with enzyme in the presence of different compounds labeled below the autoradiogram. Click here for file
