# Supplementary figures and images for: Plasmodium falciparum UvrD activities are downregulated by DNA-interacting compounds and its dsRNA inhibits malaria parasite growth
Source: BMC Biochem. 2014 Apr 3;15:9. doi: 10.1186/1471-2091-15-9 (PMC4234510; doi:10.1186/1471-2091-15-9)

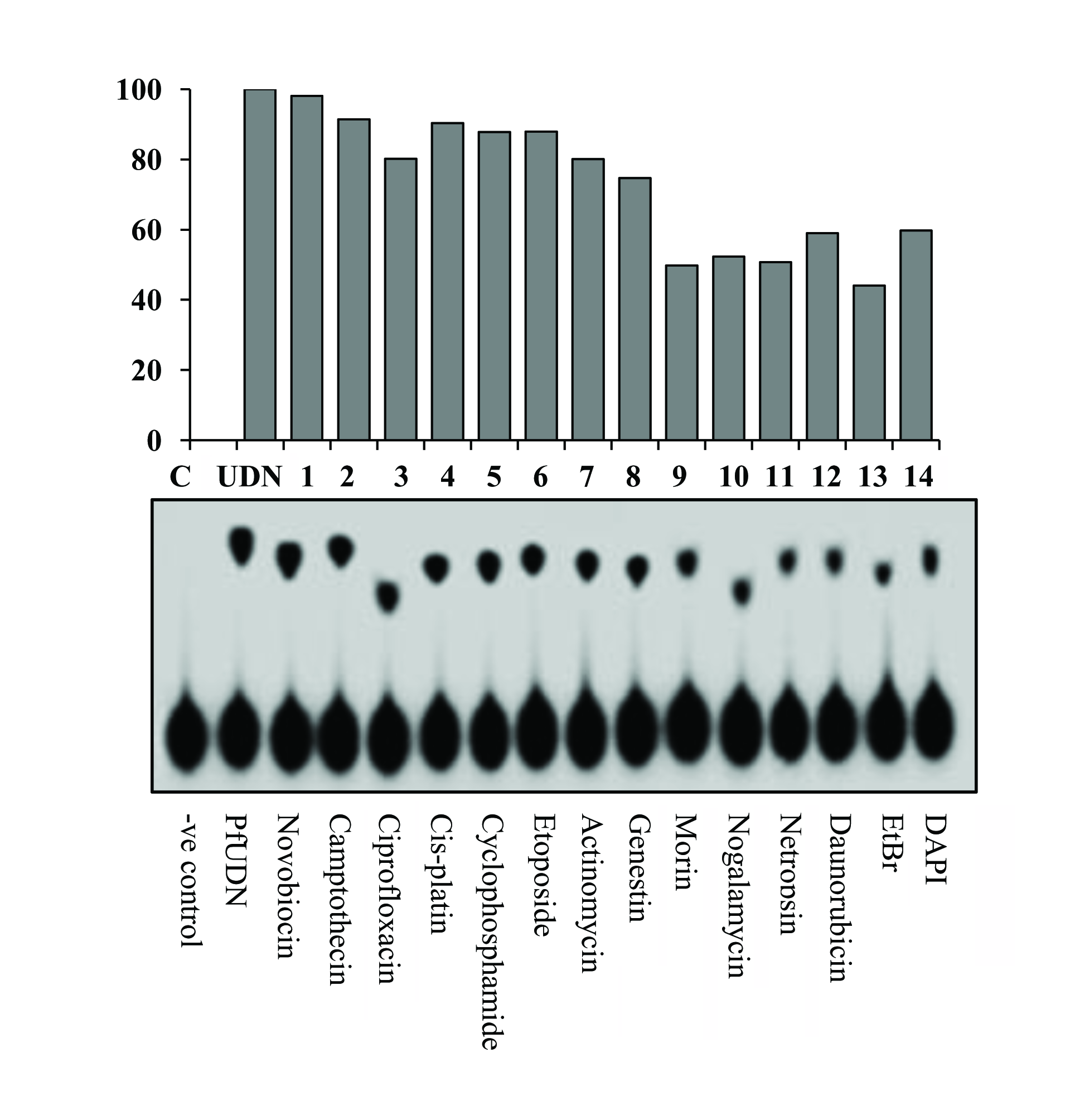

Supplement: Additional file 1 — Effect of various compounds (20 μM) on the ATPase activity in presence of 100 ng of ssDNA. ATPase assay using DNA preincubated with the compounds was done. Percent ATPase activity of PfUDN in the presence of various compounds is presented in the bar diagram corresponding to the autoradiogram. C is no enzyme control, lane UDN is control reaction of PfUDN without the addition of compound. Lanes 1-14 are the ATPase reactions with enzyme in the presence of different compounds labeled below the autoradiogram. [file 1471-2091-15-9-S1.tiff]
